# A GIS-based approach to identifying communities underserved by primary health care services—An Afghanistan case study

**DOI:** 10.3389/fpubh.2023.1209986

**Published:** 2023-09-21

**Authors:** Ramesh Nassery Mohammed, Abdullah Khawari, Jerome Aondona Shaguy, Alaa Abouzied

**Affiliations:** ^1^Health Emergency, World Health Organization, Kabul, Afghanistan; ^2^Public Health Department, Faculty of Medicine, Cairo University, Giza, Egypt

**Keywords:** primary health care, underserved and unserved populations, Afghanistan, geographical information system (GIS), health indicator

## Abstract

Afghanistan has been in an active state of conflict and war for twenty continuous years. Social services like health and education have been badly affected, facing issues such as service disruption, brain drain, and generalized instability. Health indices that provide proxy indicators for general population wellness, such as maternal health, child mortality, and immunization coverage, show that the health services available to the Afghan population are sub-optimal. Investment in social service and interventions has increased. The World Bank and the United Nations through its agencies (The World Health Organization (WHO) and United Nations' Children's Fund (UNICEF) are providing social support through targeted and strategic programs. However, the topographic and environmental realities of Afghanistan, with its broad mountain coverage, propensity toward natural disasters, and latent conflict, has made data and information gathering arduous. Since data is essential for measurement and management, the WHO Health Emergencies (WHE) information management unit at WHO Afghanistan has delivered an innovative form of data analysis, specialized and targeted at providing improved information on communities that are not adequately covered by health services. Deploying a geographical information system (GIS) approach, the WHE team has collated primary and secondary data from a combination of datasets to produce a far-reaching piece of analysis. The analysis of underserved communities in hard to reach, remote locations, provides a live, evidence-based information product. This provides a working tool that is essential to primary health programming and intervention in Afghanistan. The estimates show that approximately 9.5 million individuals in 22,181 villages across 34 provinces are underserved by primary health services. This paper is presented to explain the underpinning methodology.

## Introduction

The generalized insecurity in Afghanistan, caused by the extended state of war and exacerbated by the recent change in government, means there are substantial gaps in social services; healthcare is thus deeply affected (1). Health indices that show the general strength of a health system include immunization coverage, infant and child mortality, and maternal mortality. In Afghanistan, there Is 60% coverage for PENTA3 and Measles, Infant Mortality is at 46 per 1,000, and maternal mortality is (1) 638 per 100,000; these factors along with communicable disease susceptibility (Acute Watery Diarrhea (AWD), Measles, and Dengue Fever) show that much more investment needs to be channeled to primary health care services. Afghanistan is of largely mountainous terrain^1^, there is a significant presence of high-risk mobile populations^2^, and the last census was conducted more than four decades ago (1979). These factors make functional planning and response to health emergencies quite challenging. The identification of populations living in locations not adequately covered by primary health services is undertaken with the major objective of addressing this challenge by deploying technology and data with a creative approach. The analysis of underserved populations is undertaken to provide an overview of service gaps using a data-layered combination approach.

Afghanistan's current humanitarian development trajectory is trending downwards. The United Nations Development Program mentions that the pace of development started slowing down from 2012, with increased insecurity and instability (1). Healthcare has remained a key area of need (1). For example, Afghanistan has a severe child malnutrition burden; the United Children's Fund (UNICEF) has announced that 10 million children require humanitarian assistance^3^, while one in three adolescent girls in Afghanistan is anaemic. Due to the terrain in Afghanistan-−80% of the land is described as mountainous^1^–and with populations scattered amongst several pockets of settlements, providing stationary primary health services to adequately cover these populations is a challenge, for which high-quality data interpretation is the most viable solution.

## Methodology

The underserved area analysis is based on a geographical information system approach. This is the most appropriate approach, as the only other scientifically credible methodology is a survey-based approach. A survey-based approach requires a large degree of resources and time. Recognizing these time and resource constraints, the health information management team has taken a GIS approach. To do so, they have used layers of collated and publicly available datasets showing the following features:

1. *Population concentrations remotely sensed and surveyed*.
2. *Land coverage*.

After the above datasets were gathered, the analysis was carried out using the geospatial model and a specialized GIS software called AccessMod.^4^ AccessMod (version 5) is a free and open-source standalone software^4^ used to model the proximity of health services to target populations.

Accessing distance to care in this context estimates the portion of the target population for which services are not accessible due to service shortfalls in terms of capacity and other causal factors. These include factors that can be described in both a human and material resources sense. Distance is thus factored and calculated as a barrier to improved health outcomes.

This analysis has combined population concentrations, health facility presence (secondary and tertiary institutions have been excluded), topography, and social profiling, factoring all the dimensions to determine where additional services are most needed. The specific data sources per each data dimension are listed below:

1. *Humanitarian Data Exchange (HDX). For Rivers and roads* ^5^
2. *Afghan Geodesy and Cartography Head Office (AGCHO) for admin boundaries*.^6^
3. *Esri landcover 10 m resolution from 2021: Landcover*.^7^
4. *Digital elevation mode*.^8^

Table 1 shows a list of the datasets combined and loaded into AccessMod.

**Table 1:** Description of AccessMod input dataset.

**Data type**	**Name**	**Description**
*Raster*	Population distribution grid	Spatially explicit distribution of the population over the area.
	Land use grid	Spatial distribution of the different categories of land use on which traveling speed may be different. This grid can be combined in AccessMod with additional landscape elements (e.g., Roads or rivers) to obtain the final landcover grid.
	Digital elevation model (DEM)	Altitude distribution is used to derive slopes and correct traveling speeds in the case of anisotropic Movements.
*Vector*	Health facility location	This point shapefile contains the geographic locations of the existing network of health facilities. Its attribute table contains the population coverage capacity and the most detailed information regarding health services' availability.
	Road network	This line shapefile contains the road network. Different types of roads can be incorporated and combined with the land use grid.
	Barriers to movement	Both line and polygon shapefiles can be treated as complete barriers to movement and can be integrated into the final landcover grid.
*Tabular*	Traveling scenario	This file defines the traveling speed and the mode of transportation (e.g. walking or motorized) of each landcover.

**Figure 1:**
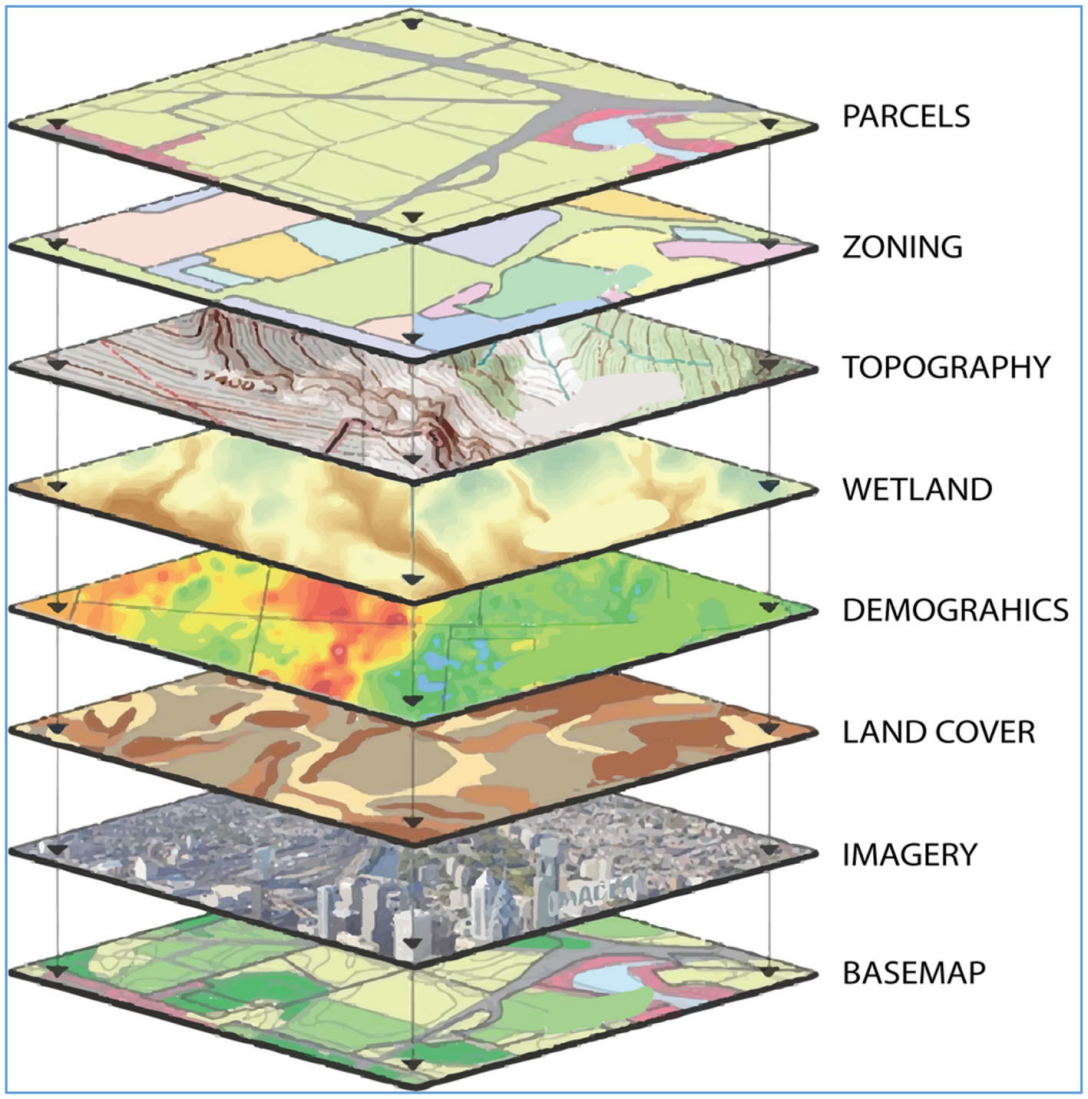
Geographical layers.

An illustration of the deployed layers is seen in Figure 1 below.

The quality of the output is directly symmetrical to the quality of the constituent datasets. The depth of the datasets and validation process undertaken to qualify them for inclusion in this analysis is described per dataset below.

### Population data

There is no current national population census in Afghanistan; the last census was conducted in 1979, and planning figures currently used in Afghanistan are based on a growth factor with the 1979 census as base data. As such, deciding on the most credible, evidence-backed population dataset presented a first-line challenge. This was resolved by settling on population figures from the world pop dataset^9^; worldMpop deployed a methodology that is the most comprehensive and so therefore was considered the most robust.

“*WorldPop researchers worked closely with the Afghan national statistical offices to integrate satellite-based mapping of all residential compounds in the country with other geospatial datasets and recent small area population enumeration in a spatial statistical modeling framework. New population estimates were produced at national, provincial, district, enumeration areas, and 100 m grid cells for the country, with associated measures of uncertainty. Cross-validation showed strong predictive ability, particularly at district and provincial scales*.”^10^

Population figures were also collated for the community level.^11^ This was necessary in order to triangulate data for greater validity and more importantly to get better estimates of catchment area populations. The source for the village- and settlement-level populations is the MISTI (Measuring Impact of Stabilization Initiatives).

“*This is the largest and most comprehensive evaluation of stabilization interventions ever undertaken by USAID*. *The MISTI dataset contains five semi*−*annual iterations or ‘waves*′* of surveys conducted by Management Systems International* (*MSI*) *from September *2012* through November *2014* to assess the impact of USAID projects on stability and resilience at the district and village levels in Afghanistan*.″11

**Figure 2:**
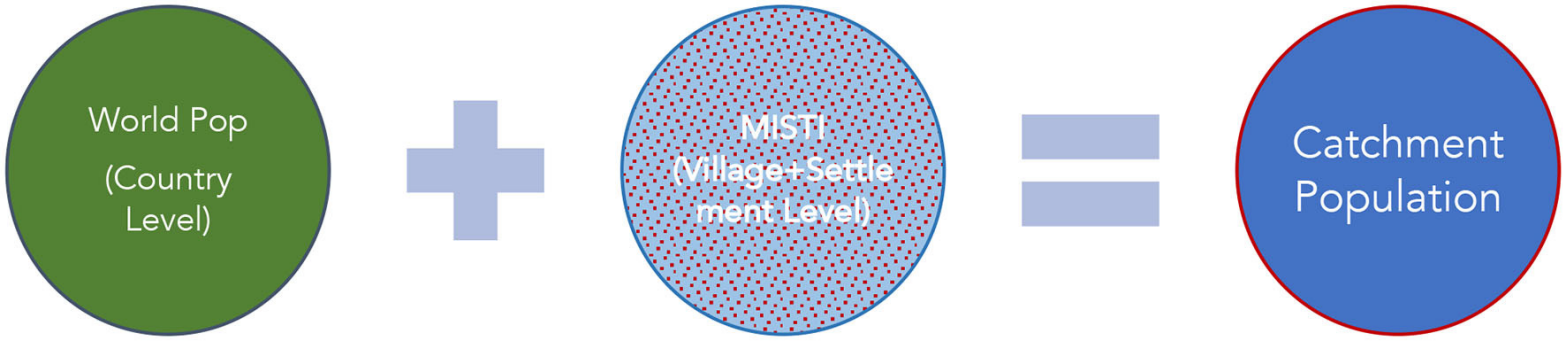
Datasets used in catchment population estimation.

Population data used for the white area analysis Figure 2.

**Figure 3:**
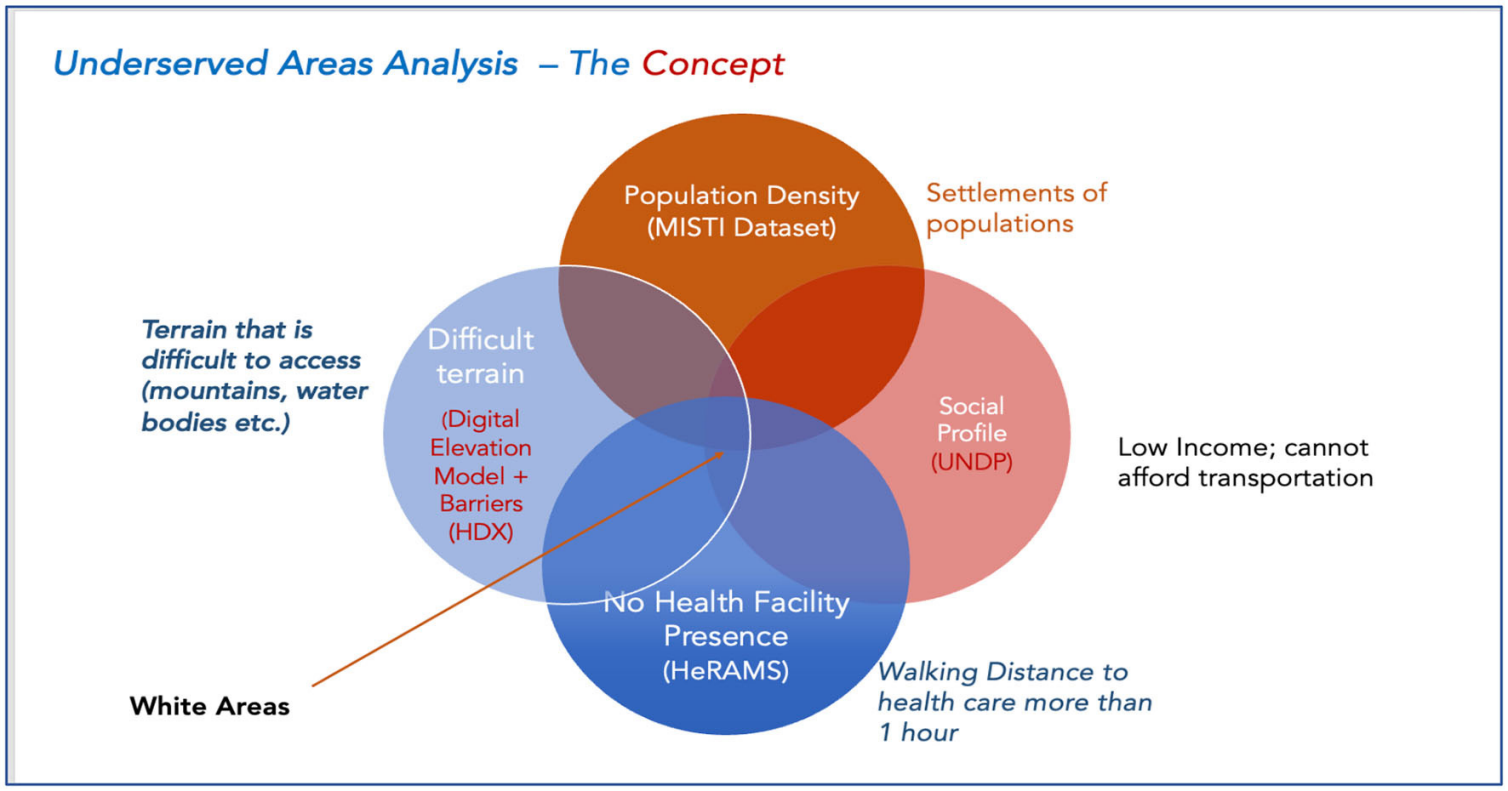
An illustration of the white area concept.

### Health facility presence data

Health Facility Presence is the key factor determining the adequacy of service availability. Data on health resource status and availability is collected in the Health Resources Availability Monitoring software (HeRAMS). HeRAMS is self-reported and routinely updated by field-level implementing partners and is currently the most comprehensive dataset of health resources available in Afghanistan. Health presence and resource availability at current levels show there are a total of 4,242 health facilities, consisting of Table 2.

**Table 2:** Desegregation of health facilities by type.

*1,286 Secondary Health Centers*
*1,003 Basic Health Centers*
*451 Comprehensive Health Centers*
*720 Mobile Health Teams*
*320 Family Health Houses*
*94 District Hospitals*
*92 First Aid Trauma Points (FATPs)*
*32 Provincial Hospitals*
*27 Specialist Hospitals*
*16 Regional Hospitals*
*5 National Hospitals*
5 *196 Centers (classified as others)*

### Social profiling data

The core determinant of vulnerability in Afghanistan's population is the earning status of each citizen. This by extension determines the access they may have to health care. The data source for this dimension of the analysis is the UNDP survey from 2021^12^, in which it is estimated that 97% of Afghans were at risk of poverty in 2022. When that data is cross-analyzed with remote sensing data, the volume of the population profiled as socially disadvantaged and occupying remote areas is marked as underserved. Below is an illustration of the combined datasets in the underserved area analysis.

### Analysis

All datasets were gathered in one unified geodatabase and imported into Access-MOD; this is the gold standard in terms of dataset management. The next step was processing. The collected data was rigorously validated through quality controls i.e., data cleaning (value field standardization, outlier elimination, gap elimination, and consistency checks). This was repeated and cross-applied throughout the data flow framework. Other checks for reliability, completeness, and external validity were applied. After the data was consolidated into Access Mod, the measures of access were then calculated based on the following criteria:

1. Measure physical accessibility to health care i.e., the travel time by walking distance of 1 hour or four kilometers based on the SPHERE framework i.e., between each community or settlement and the nearest health facility. In this analysis, 4,242 health facilities meeting the definition of basic package of health services (BPHS) were considered, excluding all hospitals. Hospitals were excluded because the metric of health care coverage defined under the SPHERE Framework is pegged at the primary health care level.
2. Estimate geographical coverage (a combination of availability and accessibility coverage) of an existing health facility's catchment area.

## Results and findings

There are more than 4,000 public health facilities operating in Afghanistan. However, due to the scattered population, geographical terrain, and environmental realities, these facilities are unevenly distributed within and among the provinces and districts. As a result of this, more than 25% of the Afghan population resides in areas where primary healthcare services are not accessible within 1-h walking distance from their homes. In actual practice, a health facility might be, in certain cases, within half a kilometer of the village, yet it is inaccessible due to physical barriers like deep or fast-flowing rivers, snow cover, or precipitous terrain.

In addition to complex emergencies, geographical barriers also hinder access to healthcare for many, particularly for those who reside in remote, hard to reach areas. The WHO carried out a geospatial analysis of underserved areas in Afghanistan in September 2022; substantial improvements in the health system and the health status of the people of Afghanistan have been achieved in recent years. Despite these improvements, Afghanistan's health indicators are near the bottom of international indices (2), and are lower than any other country in the region.

**Figure 4:**
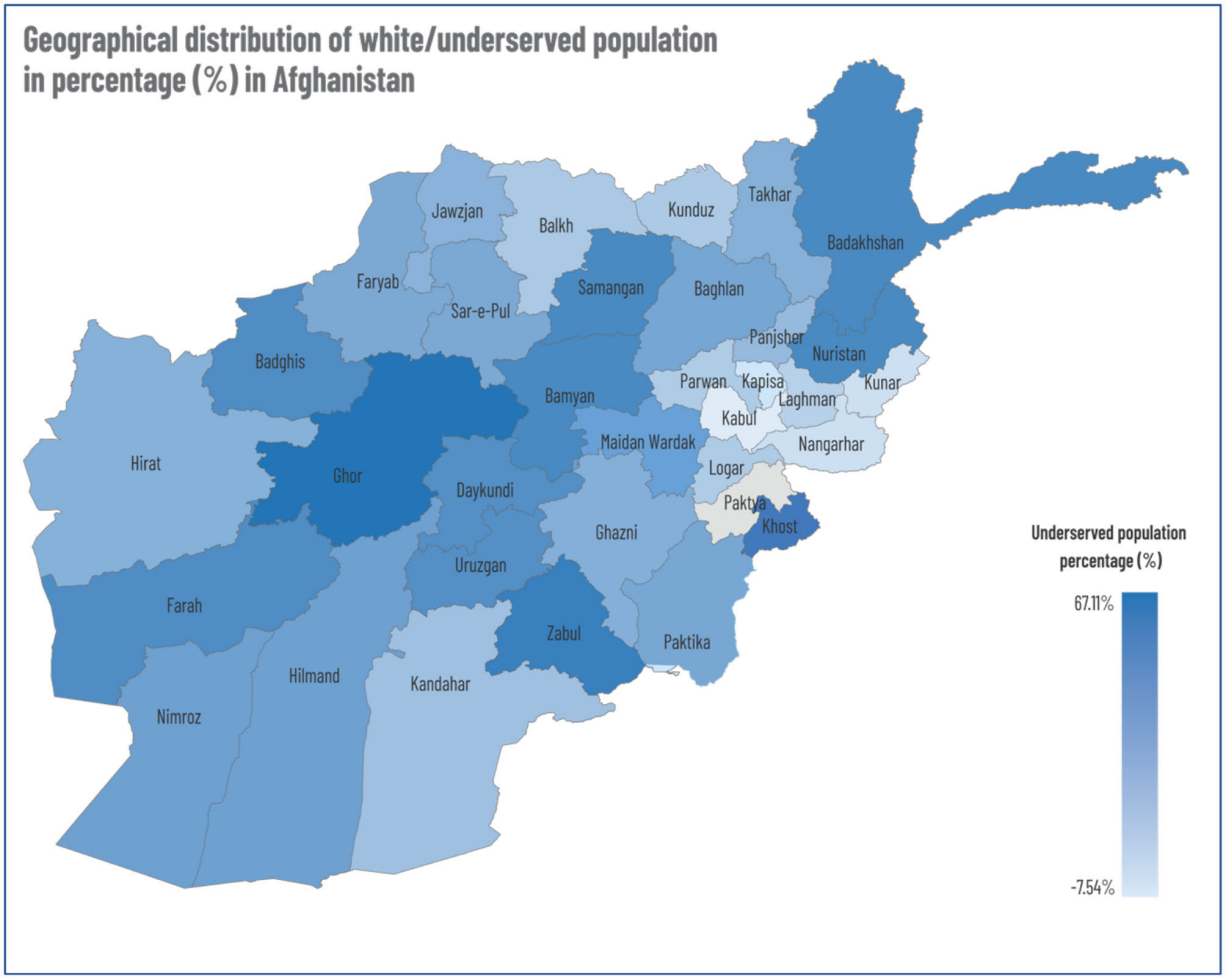
Geographical distribution of white/underserved population in percentage (%) in Afghanistan.

The findings indicate that out of the 38.2 million population, 28.7 million (75%) were covered by existing health facilities, however, 22,181 villages were identified as underserved areas with a population of 9.5 million (25%) in 34 provinces residing in areas where primary healthcare is not accessible within 1 h's walk from their homes or camps. More details on underserved population distribution across the provinces is found in Figure 4 below.

In order to disseminate findings and make the underserved area information more accessible and, by extension, actionable, the WHO health Information management team developed an online health Information portal for products and information. The analysis of underserved areas is shared through a GIS online dashboard on this portal, where health partners and stakeholders can easily access the information and take the necessary actions in order to reach the most vulnerable population using a credible evidence base.

In Figure 4 below, concentrations of underserved populations are illustrated on Afghanistan's administrative map.

### Limitations

This analysis has deployed the best available resources and tools. However, due to the constraints of the working environment, some limitations are acknowledged and declared herewith:

1. **Population data**: as has been acknowledged, Afghanistan has been in a prolonged state of war. As such, the last nationwide census was conducted in 1979, despite the fact that these should be conducted every 10 years. The absence of a national census has left a credibility gap—as key stakeholders tend not to agree on the which dataset represents the most accurate projection of the total population. This analysis was based on world pop data, which is a robust projection. Nonetheless, the absence of a national census is a limitation. This is also true of the settlement data, where MISTI data collected in 2014 is the only source of settlement population counts.
2. **Facility coordinates:** facility presence is a baseline requirement for this analysis. A high level of effort was deployed toward collecting high-quality data on facility locations. Nonetheless, this data was collected on a self-reporting database. In some instances, the pace of collection (collected data undergoes validation and quality checks) was not in pace with the update cycle incorporated into the underserved area analysis.

### Dissemination

The framework for disseminating this analysis and directing its power to relevant stakeholders has been conducted through information management products and through the launch of a public portal at https://dashboard.whe-him.org/index.php/maps-3/.

### Further analysis

In future versions of this analysis, additional layers and dimensions of data will be added. These include new features such as location radius catchment calculation (from any point on the map).

## Data availability statement

The datasets presented in this study can be found in online repositories. The names of the repository/repositories and accession number(s) can be found below: https://dashboard.whe-him.org.

## Author contributions

RM and AK are the co-technical lead in producing this paper and provided the core text and resources in the methodology section. JS is the technical lead of the HIM team and he has collated the technical data in the methodology and written up the overall narrative of the report. AA is the team lead of the World Health Emergencies section and this analysis has been done under his facilitation and supervision. He is a public health professor and has provided the editorial oversight to ensure technical validity and rigor. All authors contributed to the article and approved the submitted version.

## Footnotes

^1^ https://peakvisor.com/adm/afghanistan.html

^2^ https://dtm.iom.int/afghanistan

^3^ https://www.unicef.org/afghanistan/nutrition

^4^ https://www.accessmod.org/

^5^ https://data.humdata.org/dataset/hotosm_afg_roads

^6^ https://www.loc.gov/item/lcwaN0015981/

^7^ https://livingatlas.arcgis.com/landcover/

^8^ https://www.earthdata.nasa.gov/

^9^ https://www.worldpop.org/geodata/summary?id=39898

^10^ https://www.worldpop.org/case/case_studies/mapping_afg_pop/

^11^ https://2012-2017.usaid.gov/data/dataset/2cafcb62-00da-4f83-ac7b-471bccf8528f

^12^ https://www.undp.org/press-releases/97-percent-afghans-could-plunge-poverty-mid-2022-says-undp
